# Circular RNA circ‐RCCD promotes cardiomyocyte differentiation in mouse embryo development via recruiting YY1 to the promoter of MyD88

**DOI:** 10.1111/jcmm.17336

**Published:** 2022-06-12

**Authors:** Yiwen Liu, Jianfang Gao, Min Xu, Qianqian Zhou, Zhongxiao Zhang, Jiaxin Ye, Rui Li

**Affiliations:** ^1^ Department of Pediatrics Tongren Hospital Hongqiao International Institute of Medicine Shanghai Jiao Tong University School of Medicine Shanghai China; ^2^ Department of Nursing Tongren Hospital Hongqiao International Institute of Medicine Shanghai Jiao Tong University School of Medicine Shanghai China; ^3^ Department of Cardio‐Thoracic Surgery Nanjing Drum Tower Hospital The Affiliated Hospital of Nanjing University Medical School Nanjing China

**Keywords:** cardiomyocyte differentiation, circ‐RCCD, congenital heart disease, MyD88, YY1

## Abstract

Congenital heart disease (CHD) is the most common birth defect, affecting approximately 1% of live births. Genetic and environmental factors are leading factors to CHD, but the mechanism of CHD pathogenesis remains unclear. Circular RNAs (circRNAs) are kinds of endogenous non‐coding RNAs (ncRNAs) involved in a variety of physiological and pathological processes, especially in heart diseases. In this study, three significant differently expressed circRNA between maternal embryonic day (E) E13 and E17 was found by microarray assay. Among them, the content of circ‐RCCD increases with the development of heart and was enriched in primary cardiomyocytes of different species, which arouses our attention. Functional experiments revealed that inhibition of circ‐RCCD dramatically suppressed the formation of beating cell clusters, the fluorescence intensity of cardiac differentiation marker MF20, and the expression of the myocardial‐specific markers CTnT, Mef2c, and GATA4. Next, we found that circ‐RCCD was involved in cardiomyocyte differentiation through negative regulation of MyD88 expression. Further experiments proved that circ‐RCCD inhibited MyD88 levels by recruiting YY1 to the promoter of MyD88; circ‐RCCD inhibited nuclear translocation of YY1. These results reported that circ‐RCCD promoted cardiomyocyte differentiation by recruiting YY1 to the promoter of MyD88. And, this study provided a potential role and molecular mechanism of circ‐RCCD as a target for the treatment of CHD.

## 1 INTRODUCTION

Congenital heart disease (CHD) is a structural birth defect of the heart or great vessels induced by multi‐factors.^1^, ^2^ As the most common congenital disease and the leading cause of foetal mortality, CHD affecting approximately 1% of live births.^3^, ^4^ Although the molecular culprit of CHD pathogenesis remains unclear, perturbation of the normal program of cardiac development can be regarded as the root cause of CHD.^5^ Abnormal anatomical structure in the process of heart development often leads to heart malformations, such as loopholes in some valves or interventricular septum, transposition of the great arteries, and abnormalities in the relationship between the left and right sides of the heart, which will lead to CHD.^5^ Recently, the improved modern medical and surgical treatment has increased CHD prevalence in older individuals and has exposed comorbidities that impair quality of life and life expectancy.^6^ Therefore, it is of great significance to study the underlying pathogenesis of CHD and formulate novel strategies to prevent CHD.

Circular RNAs (circRNAs) are kinds of endogenous non‐coding RNAs (ncRNAs), which are closed‐loop formed by the connection of the 5′ and 3′ ends.^7^ Compared with linear ncRNAs, the circular structure not only improves the stability of circRNAs but also enhances their miRNA/protein binding ability.^8^, ^9^ Previous studies have demonstrated that circRNAs are highly expressed in low‐proliferating cells such as cardiomyocytes and are more highly expressed in the foetal stage of development.^10^ Besides, Studies have shown that circRNAs play a vital role in the pathophysiology of cardiovascular disease or act as diagnostic biomarkers.^11^ For example, circRNA_000203 alleviated cardiac hypertrophy by inhibiting miR‐26b‐5p/miR‐140‐3p/GATA4 axis^12^; three dysregulated circRNAs (hsa_circRNA_004183, hsa_circRNA_079265 and hsa_circRNA_105039) were identified in paediatric patients with CHD.^13^ Exciting evidence indicates that the expression of circRNA in human cardiomyocyte differentiation and disease model systems is highly dynamic,^14^ but there is no in‐depth exploration.

In this study, three significant differently expressed circRNA (circ‐0000865, circ‐0000499 and circ‐0000804) between maternal embryonic day (E) E13 and E17 was found by microarray assay. Among them, the content of circ‐0000804 increases with the development of the heart and is enriched in primary cardiomyocytes of different species, which arouses our attention. To facilitate follow‐up research, we named circ‐0000804 as the related circRNA of cardiac development (circ‐RCCD) according to its potential function. Further experiments revealed that inhibition of circ‐RCCD dramatically suppressed the cardiomyocyte differentiation; Mechanism studies have found that circ‐RCCD inhibits MyD88 levels by recruiting YY1 to the MyD88 promoter, thereby promoting cardiomyocyte differentiation.

## 2 MATERIAL AND METHODS

### 2.1 Animal experiments

The animal project was approved by the Animal Care and Use Committee of Shanghai Tongren Hospital (2021‐039‐01). Female C57BL/6 mice were purchased from Jiangsu Animal Laboratory. The mice had free access to sufficient food and drinking water under a 12:12 h Light/Dark cycle. For the embryonic stage, the day of positive vaginal plug was regarded as maternal embryonic day (E) E0. For the postnatal stage, the day of birth was regarded as postnatal day (P) P0. Pregnant female mice and neonatal mice were euthanized by intraperitoneal injection of 200 mg/kg 2% pentobarbital sodium (Sigma) at E13, E17, P0 and P10. Then, mouse embryos were removed in a sterile environment. Heart tissues from embryonic and neonatal mice were isolated under a microscope and stored at −80°C for subsequent study.

### 2.2 Isolation of primary myocardial cells

Primary myocardial cells were isolated from the ventricular myocardium of P10 C57BL/6 mice and SD rats. After the euthanasia, the heart ventricles were isolated under a microscope and cut into 1–2 mm^3^ pieces. Then, the heart ventricles were digested by 0.25% pancreatin (Sigma) for 5 times. The digestive solution was centrifuged at 2000 rpm for 20 min. The cell precipitation was resuspended in DMEM (Gibco) containing 10% FBS (Gibco). Besides, the primary myocardial cells were further purified by Percoll (Sigma) and centrifugation. The primary myocardial cells were maintained in DMEM containing 10% FBS, 1% penicillin–streptomycin (Sigma) at 37°C under 5% CO_2_.

### 2.3 P19 cell culture and cell transfection

The mouse embryonic carcinoma P19 cell line was purchased from Cell Bank of Chinese Academy of Sciences. P19 cells were cultured in α‐MEM medium (Gibco) supplemented with 10% FBS, 1% penicillin–streptomycin at 37°C under 5% CO_2_. To establish a cardiomyocyte differentiation model in vitro, P19 cells were maintained in α‐MEM medium supplemented with 10% FBS and 1% DMSO (Sigma). After DMSO‐induced for 0, 4, 6 and 10 days, the morphology of P19 cells was observed by an inverted microscope (Nikon).

The knockdown of circ‐RCCD (circ‐RCCD KD), knockdown of MyD88 (MyD88 KD), overexpression of circ‐RCCD (circ‐RCCD OE) lentiviral vectors were purchased from Genepharma. The sequences for circ‐RCCD KD and MyD88 KD were listed in Table S1. For circ‐RCCD OE, circ‐RCCD sequences were amplified and cloned into pcDNA3.1(+) circRNA mini vector (Genepharma). The primers were listed in Table S2. For cell transfection, NC KD, NC OE, circ‐RCCD KD, MyD88 KD and circ‐RCCD OE lentiviral vectors were transfected into P19 cells by Lipofectamine™ (Invitrogen) for 48 h according to the manufacturer's instructions.

### 2.4 Microarray assay

Microarray assay was performed to assess the expression profile of circRNAs in E13 and E17. *p* value was corrected by Benjamini–Hochberg. The differentially expressed circRNAs with *p* < 0.05 were selected.

### 2.5 RT‐qPCR assay

Total RNA was extracted from heart tissues, primary myocardial cells and P19 cells using Trizol reagent (Qiagen). Then, the RNA concentrations were detected by NanoDrop (Thermo) and reverse‐transcribed to cDNA by PrimeScript™RT kit (Takara). RT‐PCR assay was conducted by PrimeScriptTMRT‐PCR Kit (Takara) to confirm the characteristics of circ‐RCCD. RT‐PCR amplification product was analysed by 1.5% agarose gel. RT‐qPCR assay was conducted by SYBR Premix Ex TaqII (Takara) by fluorescent quantitative PCR 7500 (ABI). After RT‐qPCR reactions, U6 and β‐actin were used as internal control and 2^−ΔΔCT^ method was performed to calculate all the samples. Primers were shown in Table S3.

### 2.6 Immunofluorescence assay

Immunofluorescence assay was performed in P19 cells with 1.0% DMSO treatment at 10 days. P19 cells were washed with PBS (Gibco) and fixed with 4% paraformaldehyde (Beyotime) for 15 min. Then, cells were permeated with 0.5% Triton X‐100 (Beyotime) for 20 min. The myosin heavy chain‐specific MF20 antibody (1:50, Developmental Studies Hybridoma Bank) was incubated for 2 h and the goat anti‐mouse secondary antibody labelled with red fluorescent was incubated for 1 h. Finally, the nucleus was labelled with DAPI dye (Beyotime). MF20 positive cells were observed by a fluorescence microscope (Nikon).

### 2.7 Western blot assay

The total protein was lysed from P19 cells using RIPA lysate buffer (Beyotime). Then, the protein concentrations were detected by BCA kit (Beyotime). 40 μg of protein samples were separated by SDS‐PAGE gels, and transferred into PVDF membranes and incubated with primary antibodies MyD88 (ab133739, 1:2000, Abcam) or GAPDH (ab8245, 1:5000, Abcam) overnight. Afterward, membranes were probed with secondary antibody for 2 h. Finally, ECL detection reagents (Thermo Scientific) were used to visualize the membrane.

### 2.8 Luciferase reporter assay

Online database Jaspar was used to predict the putative binding sites between YY1 and the promoter region of MyD88. To construct luciferase MyD88‐wild‐type (MyD88‐WT) plasmid, the WT promoter sequence was inserted into pGL3‐basic vector at KPN1 and hindⅢ which was amplified by primers: forward primer: 5′‐TAGGTACCGCCCGCCACCGCCTAC‐3′, reversed primer: 5′‐GCCAAGCTTAGCCTTTTGAGA‐3′. For MyD88‐mutant‐type (MyD88‐MUT) plasmid construction, forward primer: 5′‐GCTTGGGGGCTGTTCACTTACTCCGAGGCTCTAATTCCTCTAC‐3′ and reversed primer: 5′‐GCCTCGGAGTAAGTGAACAGCCCCCAAGCAGTAAGCGGTC‐3′ were used for amplification. Then, the amplification products were digested by KPNI to eliminate the template plasmid, and the digestion products were transfected into e.coil to obtain the Myd88‐MUT‐luciferase plasmid. Subsequently, cells were co‐transfected with luciferase plasmids MyD88‐WT or MyD88‐MUT and YY1 mimic. After 48 h, cells were collected to measure the luciferase activity by Dual‐Luciferase Reporter Assay (Promega). The relative luciferase activity was normalized to Renilla.

### 2.9 CHIP assay

Cells were treated with formaldehyde (Sigma) for 10 min, and glycine (Sigma) was added to terminate the crosslinking. Then, cells were crushed by ultrasound and centrifuged at 10,000 g for 10 min. The cell supernatant was incubated with lgG (ab205719, 1:200, Abcam) or YY1 (ab245365, 1:200, Abcam) antibody overnight. Then, the immunoprecipitate was precipitated and purified. Next, 0.2 mol NaCl was added to immunoprecipitate to reverse the crosslinking. The primers for MyD88 promoter were shown in Table S3. The binding of Myd88 and YY1 was examined by RT‐PCR assay.

### 2.10 RIP assay

RIP assay was performed to assess the relationship between circ‐RCCD and YY1 using Magna RIP Kit (Millipore). Cells were transfected with circ‐RCCD overexpression lentivirus, or control lentivirus. Cells were lysed in complete RNA lysate and incubated with lgG or YY1 antibody overnight. Then, proteinase K was added to remove protein at 55°C for 30 min. The input and immunoprecipitated RNA was isolated by Trizol reagent and then assessed with RT‐qPCR analysis.

### 2.11 RNA pull down assay

The biotin‐labelled circ‐RCCD probe or oligo negative control (NC) probe was fixed on streptavidin agarose beads for 1 h. After that, the probe‐beads complex was incubated with protein‐containing cell lysate overnight. The proteins washed from beads were collected and then identified by Western blot assay.

### 2.12 FISH assay

FISH assay was used to display the location of circ‐RCCD in P19 cells by a Fluorescent InSitu Hybridization Kit (RiboBio). Cy3‐labeled circ‐RCCD probe (5′‐CGCCTGTACAACATTCAGGGTACCTTGTGGTCTACAATCCCT‐3′) was synthesized by RiboBio. In brief, cells were fixed with 4% paraformaldehyde for 15 min. After prehybridization in PBS, cells were hybridized in Cy3‐labeled circ‐RCCD probe overnight. Then, DAPI staining (Beyotime) was used to reverse stain the nuclei. The images were observed by a fluorescence microscope (Nikon).

### 2.13 Subcellular isolation

The cytoplasmic and nuclear RNA of P19 cells were isolated and purified using a RNA Subcellular Isolation kit (Active Motif) according to the supplier's instructions. In brief, cells were incubated with Lysis Buffer for 15 min and then centrifuged at 14,000 rpm for 5 min. Then, the cell supernatant was used to isolate cytoplasmic RNA and cell pellet was used to isolate nuclear RNA. The cell supernatant and pellet were added Buffer G and 70% Ethanol, separately. Using purification columns to wash and purify RNA samples.

### 2.14 Statistical analysis

All experimental data in this study were performed at least 3 times. Data were analysed using the Prism GraphPad 8.0 software and presented as means ± SEM. Two‐tailed Student's *t*‐test made comparisons between two groups. One‐way ANOVA followed Tukey's poc host was used to analyse differences among multiple groups. *p* < 0.05 was considered statistically significant.

## 3 RESULTS

### 3.1 Circ‐RCCD is up‐regulated in heart development and abundant in cardiomyocytes

**FIGURE 1:**
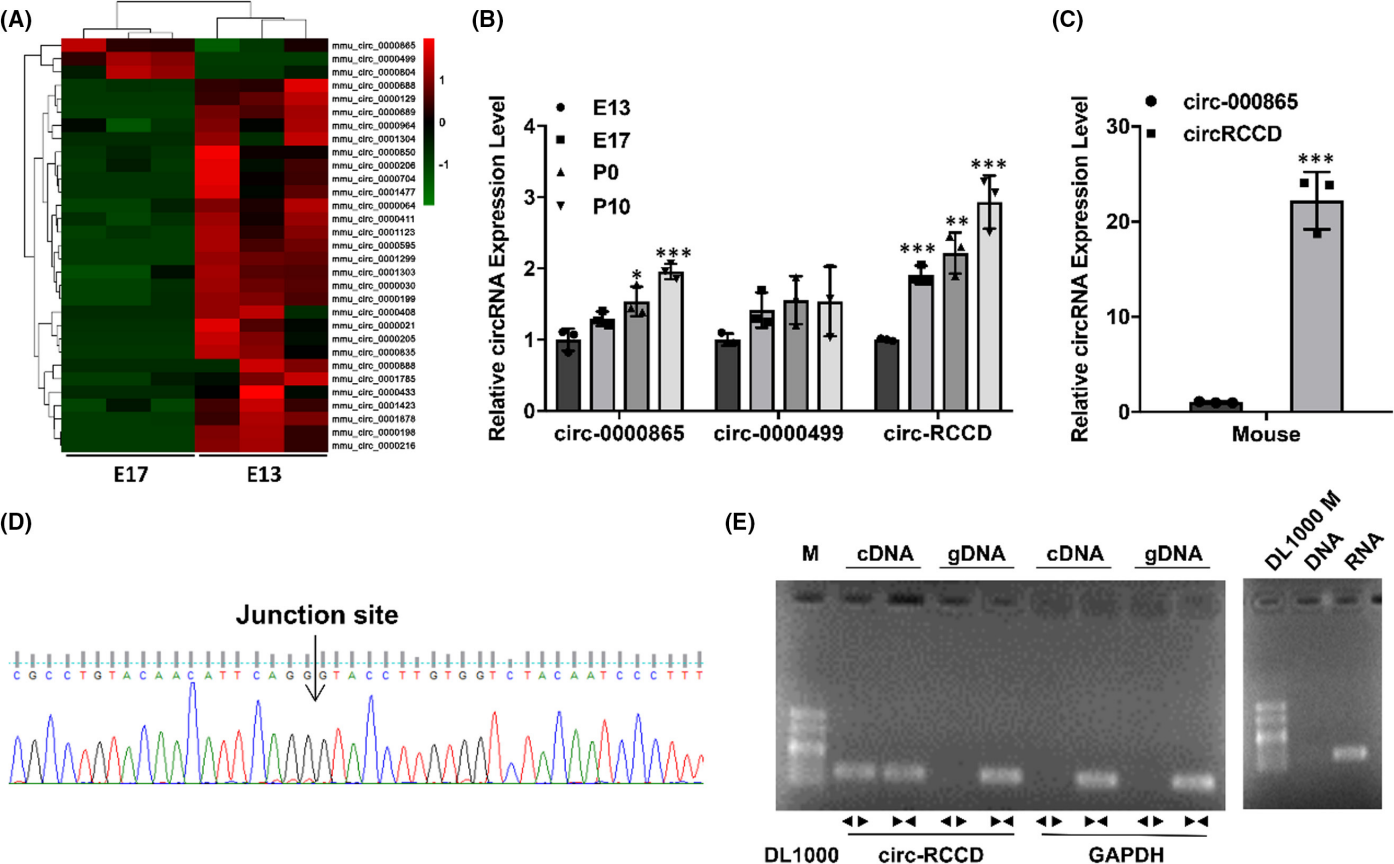
Circ‐RCCD is up‐regulated in heart development and abundant in cardiomyocytes. (A) Microarray analysis evaluated the circRNAs expression profile of E13 and E17 cardiac tissues of C57BL/6 mice. (B) The expression levels of circ‐0000865, circ‐0000499 and circ‐RCCD were detected in heart tissues at E13, E17, P0 and P10 by RT‐qPCR assay. *p < 0.05, ***p < 0.001 vs. E13 group. (C) RT‐qPCR assay detected the expression of circ‐0000865 and circ‐RCCD in neonatal cardiomyocytes from P10 C57BL/6 mice. ***p < 0.001 vs. circ‐0000865 group. (D) Sanger sequencing confirmed the back‐splice junction of circ‐RCCD. (E) The expression of circ‐RCCD was verified by PCR assay

In order to explore the potential role of circRNAs in regulating heart development, we collected heart tissues of C57BL/6 mice at E13 and E17 stages for microarray analysis. After analysing the results of circRNA microarray, we found that only circ‐0000865, circ‐0000499 and circ‐RCCD were significantly up‐regulated in E17 compared with E13 **(** *p* < 0.05, Figure 1A). The expressions of circ‐0000865, circ‐0000499 and circ‐RCCD in the hearts of C57BL/6 mice at different stages (E13, E17, P0 and P10) were detected by RT‐qPCR. It was found that the expression levels of circ‐0000865 and circ‐RCCD in E17, P0 and P10 groups were significantly higher than those in E13 (Figure 1B). In addition, primary cardiomyocytes isolated from P10 C57BL/6 mice were tested for their circ‐0000865 and circ‐RCCD content and found that circ‐RCCD was enriched (Figure 1C). These results indicate that circ‐RCCD might be involved in heart development and was worthy of further study.

The biological characteristics of circ‐RCCD was verified by Sanger sequencing and RT‐PCR assay. Sanger sequencing confirmed the back‐splice junction of circ‐RCCD, which was identical to its sequence in the circBase database (Figure 1D). Subsequently, PCR analysis confirmed that circ‐RCCD was amplified from reverse‐transcribed RNA (cDNA) by the divergent primers but not from genomic DNA (gDNA), and circ‐RCCD was amplified by RNA rather than DNA (Figure 1E).

### 3.2 Inhibition of circ‐RCCD can suppresses the differentiation of P19 cells

**FIGURE 2:**
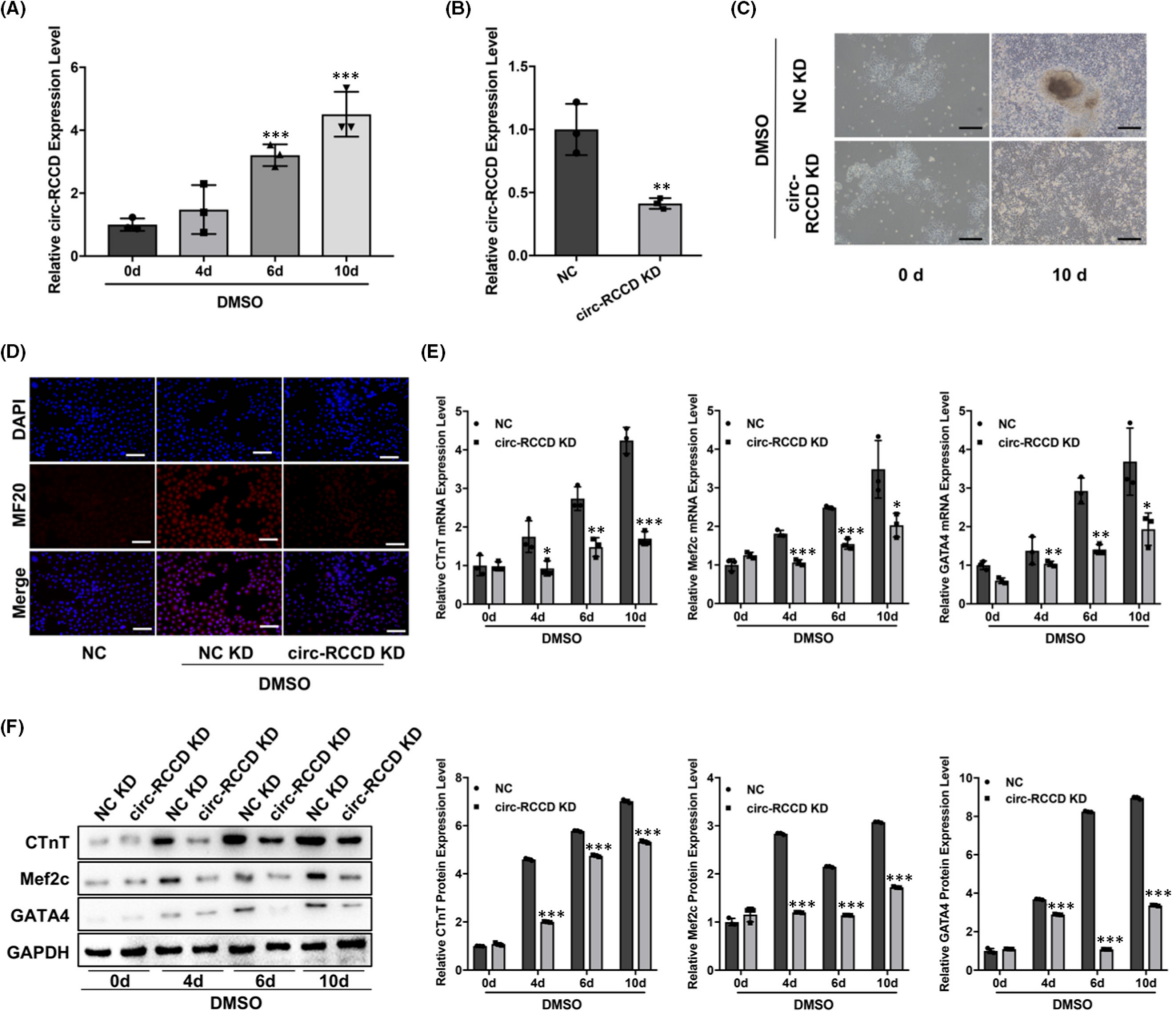
Inhibition of circ‐RCCD can suppresses the differentiation of P19 cells. (A) RT‐qPCR assay detected the expression of circ‐RCCD at 0, 4, 6 and 10 days after DMSO stimulation. ***p < 0.001 vs. 0 day group. (B) The transfection efficiency of circ‐RCCD KD in P19 cells was demonstrated by RT‐qPCR assay. **p < 0.01 vs. NC KD group. (C) The inverted microscope showed the morphology of cell P19 cells. Bar = 200 μm. (D) Immunofluorescence showed the fluorescence intensity of MF20. Bar = 100 μm. (E) RT‐qPCR and (F) Western blot assays evaluated the expression of the myocardial‐specific markers cTnT, Mef2c and GATA4 at 0, 4, 6 and 10 days after DMSO stimulation. *p < 0.05, **p < 0.01, ***p < 0.001

DMSO‐induced differentiation of P19 cells into rhythmic beating cardiomyocytes is the mainstream way to simulate myocardial differentiation. RT‐qPCR assay demonstrated that the expression level of circ‐RCCD in P19 cells were up‐regulated at 0, 4, 6 and 10 days after DMSO stimulation (Figure 2A). To explore the function of circ‐RCCD, we constructed NC KD and circ‐RCCD KD lentiviral vectors, respectively, and their transfection efficiency was shown in Figure 2B. The NC KD and circ‐RCCD KD lentiviral vectors were transfected into DMSO‐induced P19 cells. On the 10th day after transfection, the inverted microscope showed that DMSO‐induced P19 cells in NC KD group had larger, rounder, more beating cell clusters than that of circ‐RCCD KD group (Figure 2C). Besides, DMSO stimulation dramatically enhanced the fluorescence intensity of cardiac differentiation marker MF20, while inhibition of circ‐RCCD suppressed the fluorescence intensity of MF20 (Figure 2D). Furthermore, RT‐qPCR and Western blot assays were used to evaluate the expression of the myocardial‐specific markers cTnT, Mef2c and GATA4 at 0, 4, 6 and 10 days after DMSO stimulation. RT‐qPCR assay suggested that knockdown of circ‐RCCD dramatically suppressed the mRNA expression level of cTnT, Mef2c and GATA4 in DMSO‐induced P19 cells (Figure 2E). Similarly, the protein levels of myocardial‐specific markers were down‐regulated in inhibition of circ‐RCCD group during P19 differentiation (Figure 2F). In conclusion, down‐regulation of circ‐RCCD suppressed the differentiation of DMSO‐induced P19 cells.

### 3.3 Circ‐RCCD negatively regulates the expression of MyD88

**FIGURE 3:**
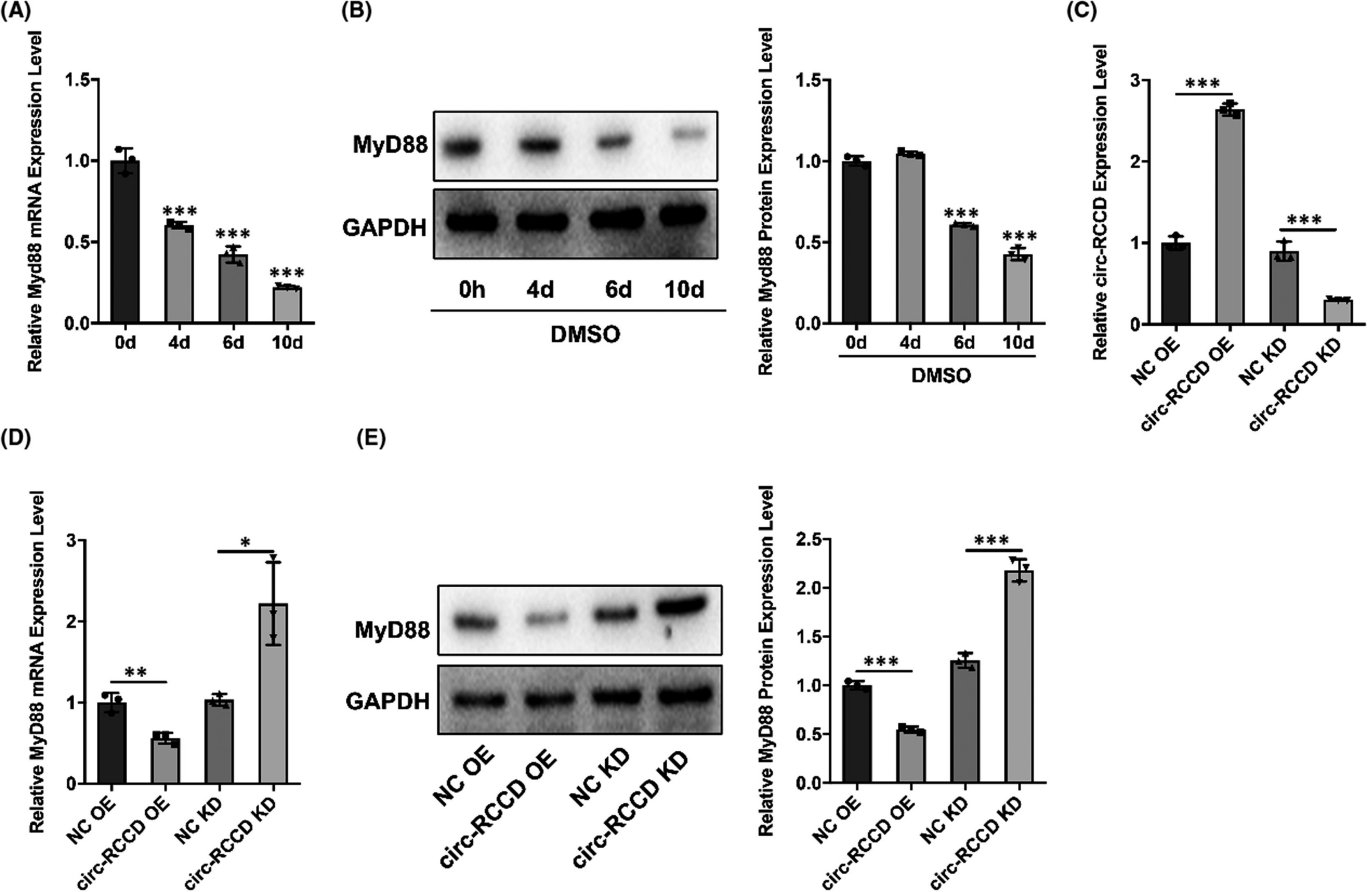
Circ‐RCCD negatively regulates the expression of MyD88. (A) RT‐qPCR and (B) Western blot assays evaluated the expression of MyD88 at 0, 4, 6 and 10 days after DMSO stimulation. ***p < 0.001 vs. 0 day group. (C) The transfection efficiency in P19 cells was confirmed by RT‐qPCR assay. (D) RT‐qPCR and (E) Western blot assays were used to evaluate the expression of MyD88 in P19 cells with NC OE or circ‐RCCD OE or NC KD or circ‐RCCD KD transfection. *p < 0.05, **p < 0.01, ***p < 0.001

It is reported that MyD88 plays a vital role in the development of heart disease.^15^, ^16^ So, we used Western blot assay to explore the expression of MyD88 in DMSO‐induced P19 cells. Results showed that both mRNA and protein expression of MyD88 in p19 cells were down‐regulated at 0, 4, 6 and 10 days after DMSO stimulation (Figure 3A,B). To further explore whether there is a regulatory relationship between circ‐RCCD and MyD88, we constructed ‘NC‐OE’, ‘circ‐RCCD‐OE’, ‘NC‐KD’ and ‘circ‐RCCD‐KD’ undifferentiated p19 cell lines through lentiviral vectors. The transfection efficiency was confirmed by RT‐qPCR assay 48 h later (Figure 3C). The results showed that overexpression of circ‐RCCD notably decreased MyD88 expression, while depletion of circ‐RCCD increased the mRNA and protein level of MyD88 (Figure 3D,E). Based on the above findings, we confirmed that circ‐RCCD can negatively regulate the expression of MyD88.

### 3.4 Inhibition of circ‐RCCD suppresses P19 cell differentiation through regulating MyD88

**FIGURE 4:**
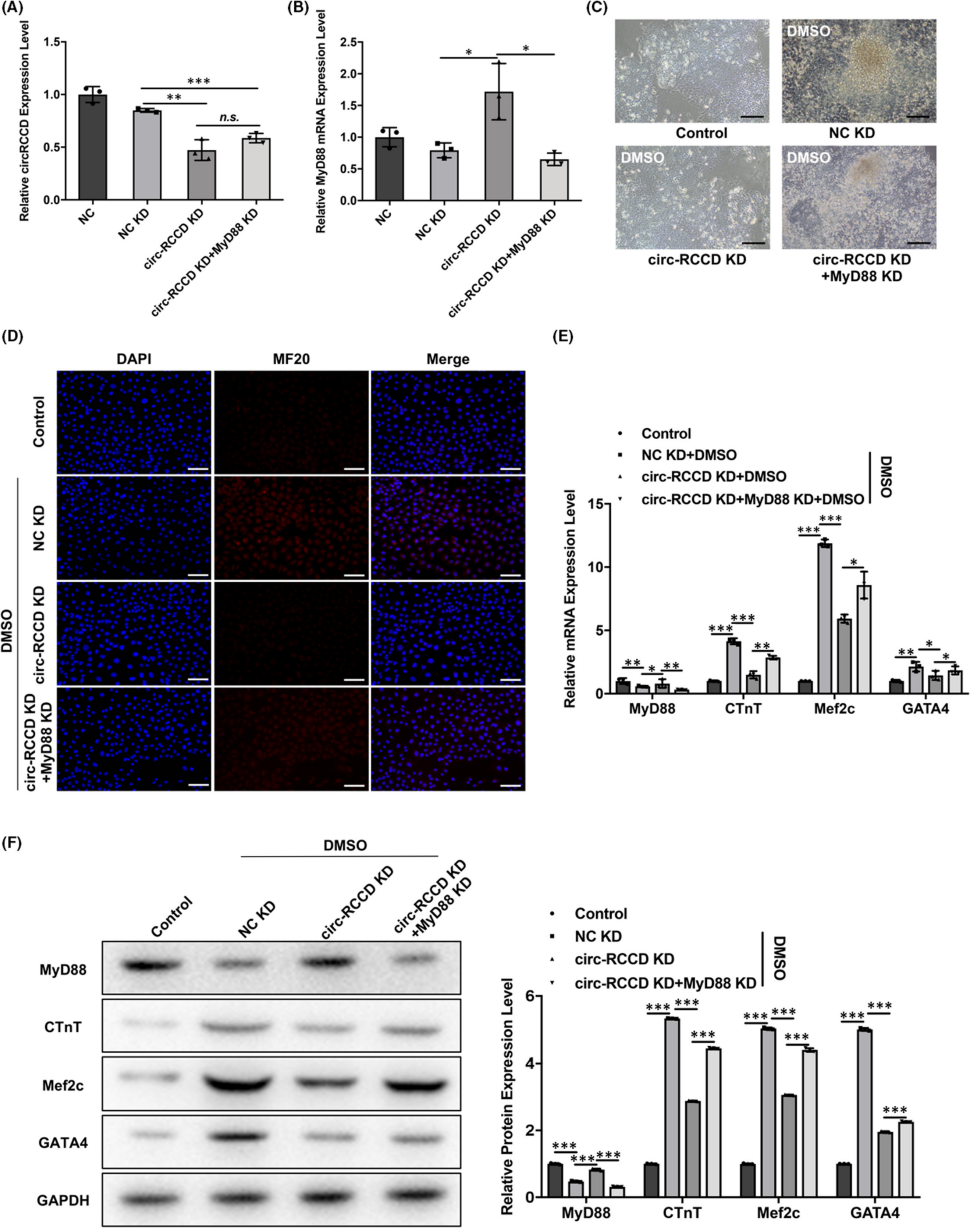
Inhibition of circ‐RCCD suppresses P19 cell differentiation through regulating MyD88. (A) circ‐RCCD and (B) MyD88 expression in P19 cells with circ‐RCCD KD and MyD88 KD co‐transfection were detected by RT‐qPCR assay. (C) The inverted microscope showed the morphology of P19 cells. Bar = 200 μm. (D) Immunofluorescence showed the fluorescence intensity of MF20. Bar = 100 μm. (E) RT‐qPCR and (F) Western blot assays evaluated the expression of the myocardial‐specific markers cTnT, Mef2c and GATA4 at 0, 4, 6 and 10 days after DMSO stimulation

To clarify whether circ‐RCCD regulates cardiomyocyte differentiation by regulating MyD88 expression, we constructed ‘NC KD’, ‘circ‐RCCD KD’, ‘circ‐RCCD KD + MyD88 KD’ P19 cell lines by lentivirus co‐transfection technology. RT‐qPCR assay revealed that circ‐RCCD expression was decreased in ‘circ‐RCCD KD’ and ‘circ‐RCCD KD + MyD88 KD’ group compared with NC KD (Figure 4A). Moreover, depletion of circ‐RCCD increased the expression level of MyD88 mRNA and protein compared with NC group, but the expression of MyD88 were suppressed in ‘circ‐RCCD KD + MyD88 KD’ group (Figure 4B,F). Functionally, the inverted microscope showed a significantly restrained DMSO‐induced beating cell clusters in ‘circ‐RCCD KD’ group; however, silencing of MyD88 abrogated this reduction (Figure 4C). And, the fluorescence intensity of MF20 was impaired by circ‐RCCD down‐regulation, while the fluorescence intensity was returned when P19 cells co‐transfection with circ‐RCCD KD and MyD88 KD (Figure 4D). Subsequently, the inhibitory role of circ‐RCCD KD on the expression of myocardial‐specific markers cTnT, Mef2c and GATA4 was abrogated by MyD88 KD (Figure 4E,F). All these data suggested that circ‐RCCD participated in cardiomyocyte differentiation through reducing MyD88 expression.

### 3.5 Circ‐RCCD interacts with transcription factor YY1 to reduce MyD88 expression

**FIGURE 5:**
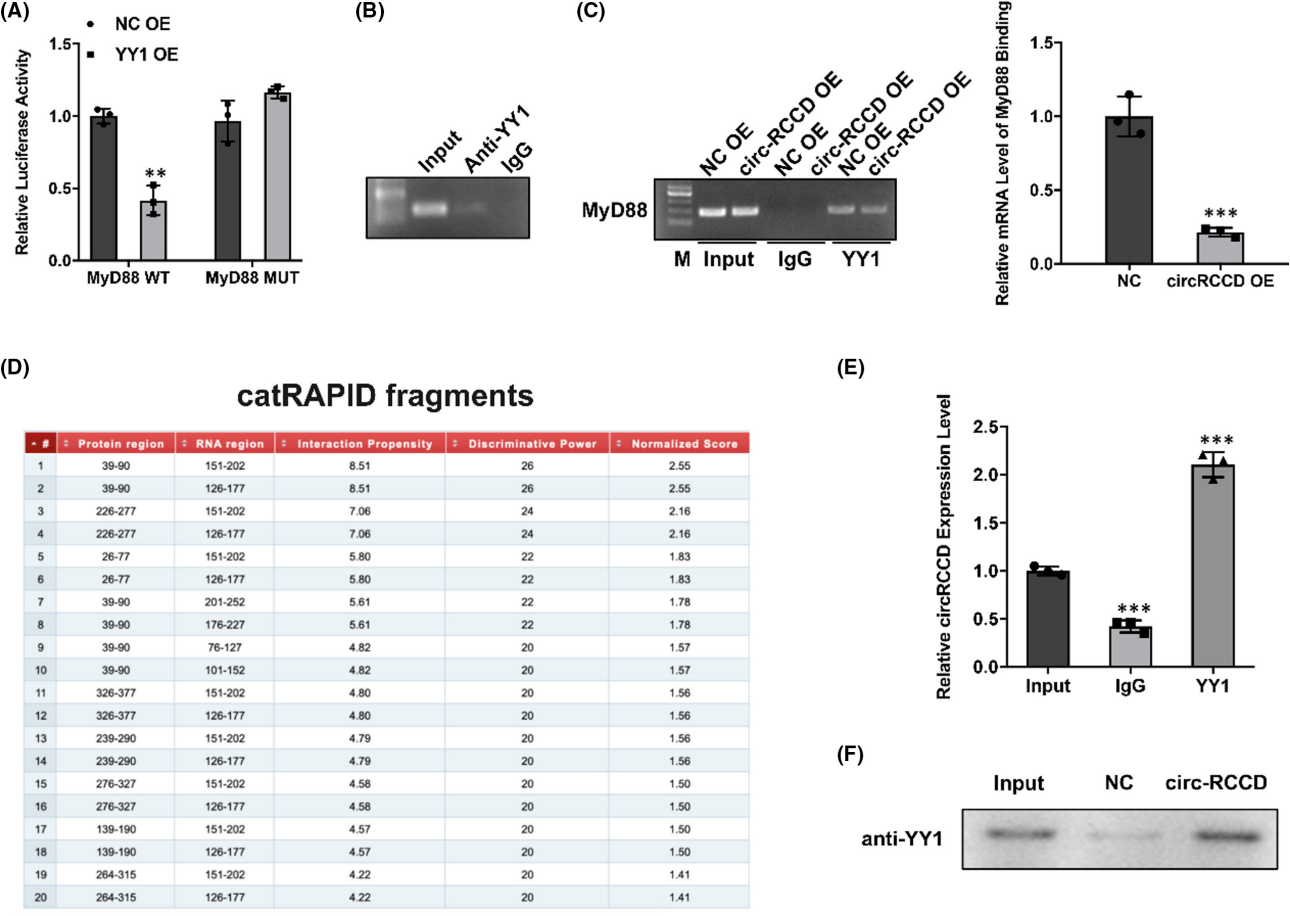
Circ‐RCCD interacts with transcription factor YY1 to reduce MyD88 expression. (A) Dual‐luciferase assay assessed the luciferase activity between YY1 and MyD88. **p < 0.01 vs. NC OE group. (B) CHIP and (C) RIP assays further confirmed the binding between YY1 and MyD88. ***p < 0.001 vs. NC OE group. (D) catRAPID showed that circ‐RCCD could bind to MyD88. (E) RIP and (F) RNA pull down assays identified the binding sites between circ‐RCCD and MyD88. ***p < 0.001 vs. Input group

Recently, studies have reported that Ying‐yang 1 (YY1), a transcription factor, exerts physiological roles in heart development via regulating gene expression.^17^ Hereby, we speculate whether circ‐RCCD interacts with transcription factor YY1 to inhibit the expression of MyD88 transcript. Wild‐type and mutant vectors of MyD88 were constructed to perform dual‐luciferase assay. The result showed that overexpression of YY1 significantly diminished the luciferase activity of wild‐type MyD88 (Figure 5A). As expected, CHIP assay confirmed the binding between YY1 and MyD88 (Figure 5B). And, the result of RIP assay displayed that overexpression of circ‐RCCD impaired the mRNA level of MyD88 binding to YY1 (Figure 5C). Moreover, the online database catRAPID showed that circ‐RCCD could bind to MyD88 (Figure 5D). And, the binding sites between circ‐RCCD and MyD88 were identified by RIP and RNA pull down assays (Figure 5E,F). In conclusion, these findings unraveled that circ‐RCCD suppressed MyD88 level by recruiting YY1 to the promoter of MyD88.

### 3.6 Circ‐RCCD interacts with transcription factor YY1 to reduce MyD88 expression

**FIGURE 6:**
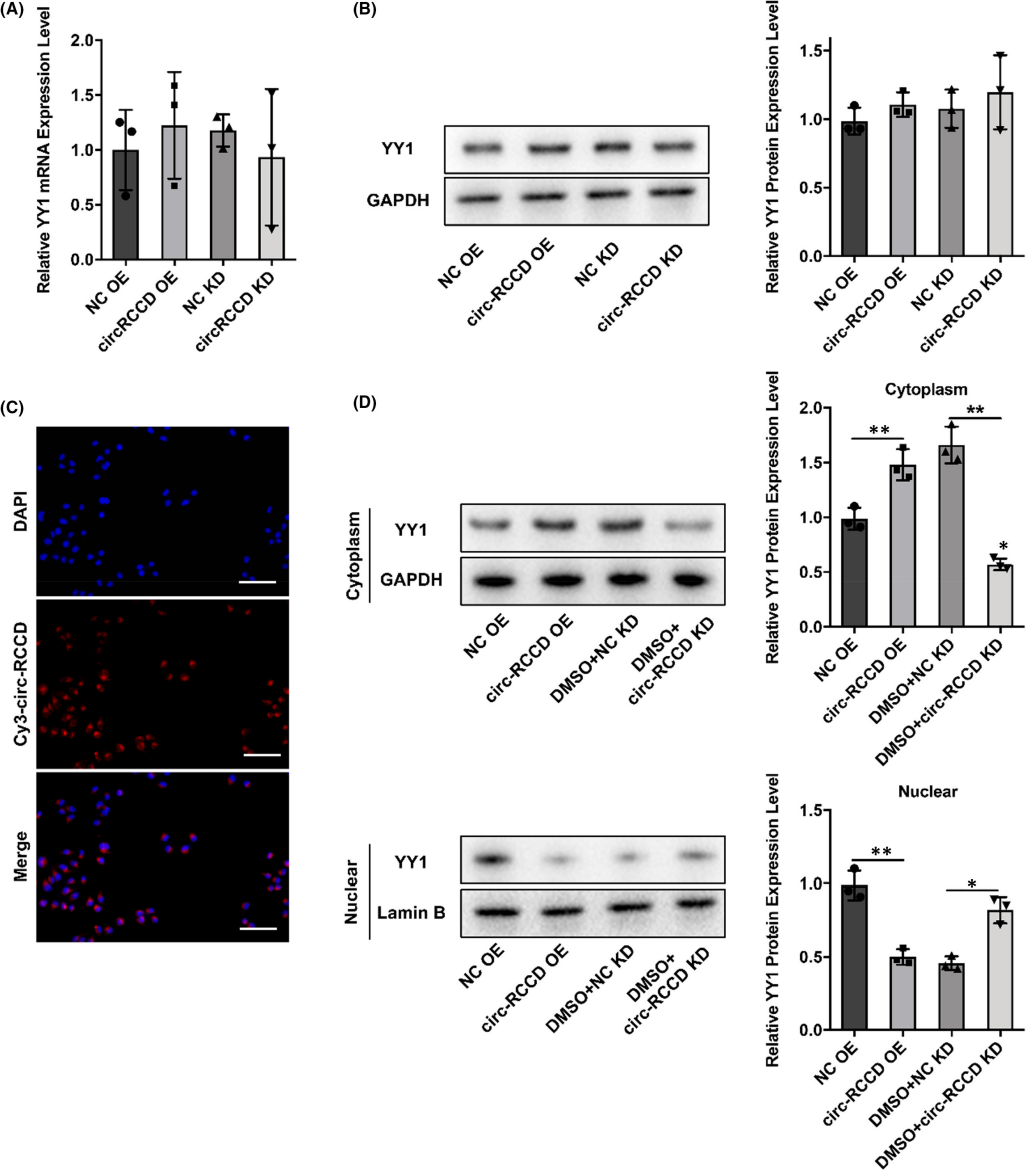
Circ‐RCCD inhibits YY1 nucleus translocation. (A) RT‐qPCR and (B) Western blot assays evaluated the expression of YY1 in P19 cells with NC OE or circ‐RCCD OE or NC KD or circ‐RCCD KD transfection. (C) FISH assay displayed that circ‐RCCD was mainly located in the cytoplasm. Bar = 100 μm. (D) Western blot assays evaluated the expression of YY1 in cytoplasm or nuclear

We then investigated how circ‐RCCD regulated YY1. RT‐qPCR and Western blot assays showed that both overexpression of circ‐RCCD and silencing of circ‐RCCD had no effect on the expression of YY1 (Figure 6A,B). Moreover, FISH assay displayed that circ‐RCCD was mainly located in the cytoplasm (Figure 6C). Then, Western blot assay showed that overexpression of circ‐RCCD increased YY1 protein expression compared with NC group in cytoplasm. Besides, YY1 protein expression was restrained by silencing of circ‐RCCD in cytoplasm. Meanwhile, overexpression of circ‐RCCD decreased YY1 protein expression compared with NC group in nuclear. YY1 protein expression was increased by silencing of circ‐RCCD in nuclear (Figure 6D). Therefore, these results indicated that circ‐RCCD inhibited YY1 nucleus translocation.

## 4 DISCUSSION

Heart is the first organ formed during embryonic development, which is regulated by multiple factors such as gene and environment. Any abnormality in the process of cardiac differentiation and morphogenesis may lead to some form of congenital heart disease.^18^ Recently, a small fluctuation in the incidence of CHD were attributed to the improvements in diagnostic methods such as echocardiography, but the overall incidence of CHD has been stable at 0.8% to 1.1%^19^; and the advanced treatment increased the prevalence of adults with CHD, it has caused a heavy burden on society at the same time.^20^ Therefore, CHD as a large, rapidly emerging global problem, is worthy of further discussion.

Emerging studies have reported that circRNAs are involved in various disease processes, including heart diseases.^11^ In 2017, Tan WL et al. detected a stage‐specific levels of circRNAs in human hearts, mouse hearts and human embryonic stem cell‐derived cardiomyocytes during the 28‐day differentiation time. Approximately 15,000 differentially expressed cardiac circRNAs were found in human hearts.^21^ Studies has reported that circSRY confers protection to hypoxic cardiomyocytes by reducing apoptosis through repression of miR‐138^22^; circ‐HRCR was located in cytoplasm, and served as miR‐223 sponge to upregulate ARC expression in cardiac hypertrophy^22^; circ‐FOXO3 was located in cytoplasm, and accelerated cardiac senescence through interacting with protein ID‐1 and transcription factor E2F1^23^; circ‐Fndc3b regulated myocardial infarction by binding with protein FUS to modulate VEGF level.^24^ Thus, starting from the perspective of circRNA regulating myocardial differentiation may provide a new method for the prevention and treatment of CHD.

In this study, we used microarray analysis to identify circRNA expression profiles at different stages of embryonic heart development. Among them, three significantly differentially expressed circRNAs (circ‐0000865, circ‐0000499 and circ‐RCCD) were screened out. Compared with circ‐0000865 and circ‐0000499, the content of circ‐RCCD increased with the development of the heart, and circ‐RCCD was enriched in primary cardiomyocytes of different species. Hence, we speculated that circ‐RCCD may play an important role in controlling cardiomyocyte differentiation.

Next, we intended to explore the molecular mechanism of heart development and cardiomyocyte differentiation in vitro. At present, three pluripotent cell lines have been isolated from living organisms, namely embryonic tumour cells, embryonic stem cells and embryonic germ cell lines.^25^, ^26^ P19 cells, derived from C3H/HE malformed neoplasms, are one of the most versatile embryonic neoplasms.^27^ Cardiomyocytes induced by differentiation of P19 cells exhibit the physiological and biochemical characteristics of early embryonic myocardium.^28^ Compared with the model established by other stem cells for inducing differentiation of cardiomyocytes, P19 cells model has the best intercellular homogeneity, which is the most beneficial for analysing the signal regulation mechanism in the process of cardiomyocyte differentiation.^29^ To establish a cardiomyocyte differentiation model in vitro, P19 cells were maintained in α‐MEM medium supplemented with 10% FBS and 1% DMSO in the study. The functional experiment further revealed that knockdown of circ‐RCCD dramatically suppressed the formation of beating cell clusters, the fluorescence intensity of MF20 and the expression of the myocardial‐specific markers cTnT, Mef2c and GATA4 in P19 cells induced by DMSO. These results indicated down‐regulation of circ‐RCCD suppressed the process of cardiomyocyte differentiation.

It is reported that MyD88 plays a vital role in the development of heart disease. For instance, MyD88 is a key signal pathway component in thyroid hormone‐induced cardiomyocyte hypertrophy.^30^ In addition, MyD88 inhibitors attenuated the infarct area during myocardial ischemia and reperfusion injury.^31^ However, the roles of MyD88 in cardiomyocyte differentiation and the expression relationship between circ‐RCCD and MyD88 remain unknown. Our study found that circ‐RCCD negatively regulated MyD88 expression, and circ‐RCCD participated in cardiomyocyte differentiation through regulating MyD88 expression. Hence, how circ‐RCCD regulates the expression of MyD88 has become the focus of our further research.

YY1 is a member of the Gli‐Kruppel family of transcription factors that play an important role in heart development.^32^ Studies have confirmed that YY1 was abundant in cardiomyocytes and was differentially expressed in many cardiovascular diseases, including CHD.^17^ Serge Gregoire et al. confirmed that the YY1 regulates Nkx2.5 expression via a 2.1‐kb cardiac‐specific enhancer, which is one of the earliest markers of differentiation of cardiac progenitor cells and is a pivotal for cardiac development.^33^

Therefore, we hypothesized that transcription factor YY1 interacted with circ‐RCCD to inhibit MyD88 expression. Consistently, the binding between YY1 and MyD88 was evidenced by dual‐luciferase and CHIP assays. Moreover, online database catRAPID and RIP and RNA pull down assays identified circ‐RCCD could bind to MyD88. It is worth noting that circ‐RCCD inhibited YY1 nucleus translocation. Thus, it was confirmed that circ‐RCCD suppressed MyD88 level by recruiting transcription factor YY1 to the promoter of MyD88.

## 5 CONCLUSION

In conclusion, circ‐RCCD is a differentially expressed circRNA identified from mouse embryonic heart tissue. The expression levels of circ‐RCCD increased with the development of the heart, and circ‐RCCD was enriched in primary cardiomyocytes of different species. Functional experiments confirmed that circ‐RCCD has the ability to promote myocardial differentiation. Further, mechanism experiments reported that crosstalk of circ‐RCCD with transcription factor YY1‐mediated MyD88 expression. These results may provide novel therapeutic strategies for heart development and diseases.

## AUTHOR CONTRIBUTIONS

**Yiwen Liu:** Project administration (equal); Writing – original draft (equal). **Jianfang Gao:** Validation (equal); Writing – original draft (equal). **Min Xu:** Methodology (equal); Writing – review & editing (equal). **Qianqian Zhou:** Formal analysis (equal). **Zhongxiao Zhang:** Data curation (equal); Formal analysis (equal). **Jiaxin Ye:** Conceptualization (equal); Resources (equal). **Rui Li:** Conceptualization (equal).

## CONFLICT OF INTEREST

The authors declare that the research was conducted in the absence of any commercial or financial relationships that could be construed as a potential conflict of interest.

## Supporting information

Tab S1‐S3
