# Supplementary material for: Circular RNA circ‐RCCD promotes cardiomyocyte differentiation in mouse embryo development via recruiting YY1 to the promoter of MyD88
Source: J Cell Mol Med. 2022 Jun 12;26(13):3616–27. doi: 10.1111/jcmm.17336 (PMC9258712; doi:10.1111/jcmm.17336)
Supplement: Supplementary file 1 — Tab S1‐S3 [file JCMM-26-3616-s001.docx]

**Table S1. Sequences of gene knockdown**

| **Gene** | **Sequences** (5’-3’) |
| --- | --- |
| circ-RCCD KD | TTGGTACCGGGCTGATGGAGAAAGTGAGATCAAGAGTCTCACTTTCTCCATCAGCCC GGATCCACT |
| MyD88 KD | ACCTCGCAACTGGAACAGACAAACTATCAAGAGTAGTTTGTCTGTTCCAGTTGCTT |

**Table S2. Primer sequences for circ-RCCD amplification**

| **Gene** | **Forward Primer** (5’-3’) | **Reversed Primer (**5’-3’) |
| --- | --- | --- |
| circ-0000865 | TTGGTACCGTACCTTGTGGTCTACAA | TTGAATTCCCTGAATGTTGTACAG |

**Table S3. Primer sequences of genes in RT-qPCR and CHIP assays**

| **Gene** | **Forward Primer** (5’-3’) | **Reversed Primer (**5’-3’) |
| --- | --- | --- |
| circ-0000865 | CGTGGGTTCTCTAGAGTCTGTA | CACGGCCCCGTCCTCGGCC |
| circ-0000499 | TGTAAGAATTGGTGGATCTGT | CACGGCCCCGTCCTCGGC |
| circ-RCCD | CCTAAGACCGCCCTTTGTCA | ACTTTCACTGTGGCGGAGTT |
| cTnT | TGGTGGAGGAGTACGAGGAG | CTACGTTGGCCTCCTCTGTC |
| Mef2c | CTGATGGGCGGAGATCTGAC | TTGCTGCCAGGTGGGATAAG |
| GATA4 | ATCCATCCAGTGCTGTCTGC | GCTGTTCCAAGAGTCCTGCT |
| MyD88 | AGAGCTGCTGGCCTTGTTAG | TCCTAGGGGGTCATCAAGGG |
| YY1 | AGCAGGTGCAGATCAAGACC | GGGTCTGAGAGGTCAATGCC |
| GAPDH | GAAGGTCGGTGTGAACGGAT | ACTGTGCCGTTGAATTTGCC |
| MyD88 (CHIP) | TGTAGCCAAACCCGAGACC | TCCTAGGAAGTGGGTAGAGG |
